# Antiproliferative and Pro-Apoptotic Effects of *Tuber borchii* Extracts on Human Colorectal Cancer Cells via p53-Dependent Pathway Activation

**DOI:** 10.3390/metabo15120796

**Published:** 2025-12-15

**Authors:** Emily Carinci, Serena Castelli, Laura Vitiello, Alessandro Pennesi, Antonella Amicucci, Alessandra Zambonelli, Maria Rosa Ciriolo, Vilberto Stocchi, Sara Baldelli

**Affiliations:** 1 Department for the Promotion of Human Science and Quality of Life, San Raffaele Open University, Rome, Via di Val Cannuta, 247, 00166 Rome, Italy; emily.carinci@sanraffaele.it (E.C.); serena.castelli@uniroma5.it (S.C.); laura.vitiello@uniroma5.it (L.V.); vilberto.stocchi@uniroma5.it (V.S.); 2 Aging Biochemistry Laboratory, Laboratory of Molecular and Clinical Epidemiology, IRCCS San Raffaele Roma, 00166 Rome, Italy; ciriolo@bio.uniroma2.it; 3 Department of Biomolecular Sciences, University of Urbino, 61029 Urbino, Italy; a.pennesi1@campus.uniurb.it (A.P.); antonella.amicucci@uniurb.it (A.A.); 4 Department of Agricultural and Food Sciences, University of Bologna, 40127 Bologna, Italy; alessandr.zambonelli@unibo.it; 5 Department of Biology, University of Rome “Tor Vergata”, 00133 Rome, Italy

**Keywords:** Bianchetto truffle, colorectal cancer, HCT 116 cells, apoptosis, p53 pathway, antiproliferative activity, nutraceutical potential

## Abstract

Background/Objectives: Colorectal cancer (CRC) is one of the most aggressive malignancies and has a very high mortality rate. Several studies have shown that obesity and hyperlipidemia are among the factors implicated in the onset of this disease. These factors can be modified through lifestyle changes, and diet plays a crucial role in this context. We evaluated the effects of *Tuber borchii* (*T. borchii*) fungal extracts based on experimental evidence showing that some truffles produce antioxidant, anti-inflammatory, and anticancer secondary metabolites. Methods: To this end, we treated human colorectal cancer cells (HCT 116) with various extracts of *T. borchii* at different time points and concentrations. Results and Conclusions: The results showed that the treatments caused a decrease in cell proliferation due to the induction of apoptotic cell death, as evidenced by FACS analyses. The apoptotic pathway was confirmed by the increase in the cleavage of Caspase 3 and Caspase 9. We then investigated the molecular mechanisms underlying cell death, finding increased nuclear localization of p53 and increased expression of its downstream pro-apoptotic genes, PUMA and NOXA. Among the upstream signaling events, we identify an increase in p-ERK1/2, a MAPK member involved in several antiproliferative/pro-apoptotic insults.

## 1. Introduction

Currently, colorectal cancer (CRC) is the world’s third most prevalent malignancy and the second major contributor to cancer mortality [1]. Major factors contributing to the early onset of CRC include obesity [2]. Many of these risks are related to dietary patterns and lifestyle choices that can be modified. In particular, diet is one of the most important factors in the development and prevention of CRC. Research has identified some nutrients and foods that may contribute to or, conversely, prevent this type of cancer, but studies in this area remain limited and sometimes inconsistent [3,4]. Therefore, it is crucial to identify new molecules capable of counteracting disease progression and reducing mortality.

Another important aspect to consider is the emergence of drug resistance, which represents a major obstacle to currently available therapies. In this context, the use of bioactive natural compounds as chemosensitizing agents has been explored and is considered is increasingly being explored and appears to be a promising approach in chemotherapy [5].

In recent years, scientific interest has shifted toward edible medicinal mushrooms, which generate clinically relevant metabolites or can be engineered to do so through biotechnological approaches. In particular, truffles, hypogeal fungi belonging to the genus *Tuber*, synthesize numerous secondary metabolites with diverse biological activities, including phenols and aromatic compounds (responsible for their characteristic aroma), unsaturated fatty acids, sterols, volatile sulfur compounds, and antioxidants (e.g., glutathione, polyphenols) [6]. Accumulating experimental data indicate the beneficial effects of these secondary metabolites found in truffles, which show promising antioxidant, anticancer, and anti-inflammatory effects thanks to their rich composition of phenols, flavonoids, terpenoids, polysaccharides, and steroids [7]. By scavenging free radicals and protecting cells from oxidative damage, these metabolites offer potential medicinal applications, including serving as adjuvants to chemotherapy.

In this work, we investigated in detail the biological activities of *Tuber borchii* (*T. borchii*), an ectomycorrhizal fungus of the genus *Tuber*, known for its appreciated organoleptic properties. Although most studies on this hypogenic fungus have focused on its chemical properties (nutritional and aromatic profiles), we aimed to analyze its potential anticancer activity in colorectal cancer cells. Although most studies on this hypogeal fungus have focused on its chemical characteristics (nutritional and aromatic profile), we aimed to explore its potential anticancer properties in colorectal cancer cells. Previous research describing the chemical composition of *T. borchii* has demonstrated its high nutritional value. The fungus contains both exopolysaccharides and intracellular polysaccharides. The extracted polysaccharides are β-(1–3)-glucans, known for their biomedical properties: anticarcinogenic, antimicrobial, and immunomodulatory [8]. Analyses also indicate a predominance of sterols, particularly ergosterol and brassicasterol [9], as well as the presence of unsaturated fatty acids, mainly linoleic and oleic acids, with smaller amounts of stearic and palmitic acids [10]. *T. borchii* has a soluble protein content of approximately 13% [11] and contains volatile terpenes associated with aroma [12]. Limonene, in particular, is present, albeit in modest quantities (approximately 0.42%) [13]. Another noteworthy characteristic of *T. borchii* is its significant flavonoid content [14], which contributes to its strong antioxidant activity [6].

Based on this background, in the present study, we characterized and assessed the antiproliferative and pro-apoptotic effects of different *T. borchii* ascoma extracts in colorectal cancer HCT 116 cells. Our results show that these extracts induce apoptosis through a p53-dependent signaling pathway, involving its nuclear translocation and the subsequent transcriptional activation of two key pro-apoptotic factors, PUMA and NOXA.

## 2. Materials and Methods

### 2.1. Truffle Collection and Molecular Identification

*Tuber* ascomata naturally growing in a specific regional area from Marche (Sant’Angelo in Vado and Urbania, Italy) were harvested in January 2025. After collection, truffles were gently cleaned to remove soil residues, individually labeled, and stored at 4 °C until DNA extraction. Genomic DNA was isolated from the fresh gleba tissue of each specimen following the protocol described by Paolocci et al. [15]. DNA concentration and purity were assessed using a spectrophotometer NanoDrop (NanoDrop ND-1000 Spectrophotometer, Thermo Fisher Scientific, Wilmington, DE, USA). Species identity (*Tuber borchii*) was verified by PCR using specific primers TboI (5′-TGTATGGGATGCCCTATCGGACT-3′] and TboII [5′-CTATTACCACGGTCAACTTC-3′). PCR reactions were carried out in a final volume of 25 μL, containing 1× TaKaRa Taq™ Buffer (TaKaRa Bio, San Jose, CA, USA), 0.4 µM of each primer, 200 µM of each dNTP (TaKaRa Bio, San Jose, CA, USA), 1 U of TaKaRa Taq DNA Polymerase (TaKaRa Bio, San Jose, CA, USA), and 1 µL of template DNA. Thermal cycling was performed in a Veriti™ 96-well Thermal Cycler (Applied Biosystems, Foster City, CA, USA) under the following conditions: initial denaturation at 95 °C for 5 min; 30 cycles of 95 °C for 20 s, 55 °C for 15 s, and 72 °C for 30 s; followed by a final extension at 72 °C for 7 min. The specific 397 bp band was analyzed by electrophoresis on 1.4% agarose gels stained with Midori Green Advance (Nippon Genetics, Düren, Germany) and visualized under UV light.

### 2.2. Preparation of the Ethanolic Extract

Four *T. borchii* fruiting bodies (no. 1–4) were subjected to ethanolic extraction to obtain bioactive metabolites. The extraction protocol was adapted from Saltarelli et al. (2019) [16] with minor modifications suitable for truffle tissue. The inner gleba portions (1–5 mm) from each *T. borchii* ascoma were excised with sterile scalpels and forceps, weighed to determine the fresh weight, and then dried at 60 °C for five hours. The dry weight of the four ascomata was recorded as follows: sample 1, 1.73 g; sample 2, 1.72 g; sample 3, 1.27 g and sample 4, 1.23 g. The dried tissues were ground in liquid nitrogen using a pestle and mortar, and the resulting powder was transferred to sterile 50 mL tubes for extraction with 20 mL of 80% (*v*/*v*) ethanol under gentle agitation at 4 °C overnight. The truffle extracts were centrifuged at 14,000 rpm for 15 min at 4 °C, and the supernatant was collected. The pellet of each sample was re-extracted twice with 15 mL of ethanol/water (80%, *v*/*v*) for 1 h under the same conditions. Supernatants from the three extractions from each sample were pooled and dried. At the end of the procedure, the final dried extracts of the four ascomata were recorded: sample 1, 0.028 g; sample 2, 0.098 g; sample 3, 0.038 g and sample 4, 0.032 g. Finally, they were resuspended in 1 mL of 80% ethanol (*v*/*v*). Extracts were stored at 4 °C for further use. In the further experiments, the *T. borchii* extracts were expressed as μg/mL.

The maturation stage of the fruiting bodies was evaluated following the method described by Zeppa et al. (2004) [17]. Only ascomata exhibiting a maturation degree of 1, corresponding to 6–30% of mature spores, were selected for analysis (Supplementary Figure S1).

### 2.3. Cell Cultures

Human colorectal cancer cells (HCT 116) were purchased from the European Collection of Cell Cultures (Salisbury, UK) and they were cultured in DMEM medium High Glucose (4.5 g/L) with L-Glutamine (Sial, Rome, Italy) supplemented with 10% FBS (Lonza, Basel, CH), 100 U/mL penicillin/streptomycin (Sial, Rome, Italy) at 37 °C in an atmosphere of 5% CO_2_ in air.

### 2.4. Analysis of Cell Viability and Proliferation 

After trypsinization (Trypsin, Sial, Rome, Italy), adherent and detached cells were combined, washed with PBS (Euro Clone, Milan, Italy), stained with Trypan Blue and then counted. Moreover, MTS assay kit ‘‘Cell Titer 961 Aqueous One Solution Cell Proliferation assay’’ (Promega, Fitchburg, Madison, WI, USA) was used for cell proliferation measurement.

### 2.5. Treatments

Treatments with the four extracts (four samples) from different truffles of *T. borchii* (extracts 1, 2, 3, and 4, dissolved in 80% ethanol, PanReac AppliChem, Darmstadt, Germany) were initially performed at two concentrations, 100 and 50 μg/mL, at 37 °C in culture medium.

The 100 μg/mL concentration of extracts 3 and 4 (samples 3 and 4) was selected for all experiments as it provided the most significant decrease in proliferation and apoptosis. As a control, an equal amount of 80% ethanol was added to untreated cells. For the treatments, cells were exposed to *T. borchii* extracts (3 and 4) for 24, 6, and 3 h.

Treatment with the cell-permeable pan-caspase apoptosis inhibitor Z-VAD-FMK (Sigma-Aldrich, Saint Louis, MO, USA) was performed at 10 µM. This inhibitor was added 1 h before treatment with the *T. borchii* extracts and maintained for the duration of the experiment (24 h).

### 2.6. Preparation of Nuclear and Cytosolic Extracts

Nuclear and cytosolic fractions were isolated to separate nuclear from cytosolic components. After detachment in PBS (Euro Clone, Milan, Italy), cells were centrifuged at 650× *g* for 5 min at 4 °C. The pellet was resuspended in 250–300 µL of extraction buffer containing: 0.01 M Tris-HCl pH 8.0 (VWR Chemicals, Radnor, PA, USA), 0.01 M MgCl_2_ (Sigma-Aldrich, Saint Louis, MO, USA), 0.01 M EDTA (Sigma-Aldrich, Saint Louis, MO, USA), 0.25 M sucrose (Sigma-Aldrich, Saint Louis, MO, USA), phosphatase inhibitors (0.001 M sodium orthovanadate, 0.002 M sodium pyrophosphate, 0.05 M sodium fluoride; Sigma-Aldrich, Saint Louis, MO, USA), 1% Triton X-100 (USBiological Life Science, Salem, MA, USA), 0.5 mM DTT and 1:100 protease inhibitors (Amresco, Solon, OH, USA). Samples were incubated on ice for 30 min, gently mixing every 10 min. The suspension was centrifuged at 650× *g* for 10 min at 4 °C. The cytosolic supernatant was collected, and the nuclear pellet was resuspended and washed three times with 500 µL of wash buffer (identical to extraction buffer without Triton X-100), centrifuging at 650× *g* for 10 min at 4 °C between washes. The resulting nuclear and cytosolic fractions were stored at −20 °C until further analysis.

### 2.7. Western Blot Analysis

Lysis buffer containing 10 mmol/L Tris-HCl, pH 7.4, 5 mmol/L EDTA, 150 mmol/L NaCl, (VWR Chemicals, Radnor, PA, USA), 0.5% IGEPAL CA-630 and protease inhibitors (Amresco, Solon, OH, USA) was used to resuspend cell pellet. Total protein (20 μg), cytosolic (20 μg) or nuclear protein (20 μg) was electrophoresed on 8.5%, 10% or 12% SDS-polyacrylamide gels and transferred to nitrocellulose (Bio-Rad, Hercules, CA, USA). Proteins were analyzed by the Lowry method [18]. All primary antibodies were used at a dilution of 1:1000. Their respective codes and suppliers are listed in Table 1.

The membranes were incubated with the appropriate HRP-conjugated secondary antibodies (Bio-Rad Laboratories, Hercules, CA, USA), and then protein bands were detected using a Fluorchem imaging system (Alpha Innotech Corporation-Analitica De Mori, Milan, Italy). ChemiGlow chemiluminescent substrate was used to detect the signal. Quantity One software 4.6.8 (Bio-Rad, Hercules, CA, USA) was used to perform densitometric analyses of protein bands. β-actin was used as a loading control.

### 2.8. Analysis of Apoptosis

After washing cells with PBS, they were stained with an annexin V-FITC/propidium iodide kit (Miltenyi Biotec, Bergisch Gladbach, Germany) and analyzed by LSR Fortessa X-20 instrument at 24 h after treatment with the extracts.

### 2.9. RT-qPCR Analysis

Total RNA was extracted after 24 h from treatment, using TRI reagent (Sigma-Aldrich, St. Louis, MO). The retrotranscription of one microgram of RNA was performed with M-MLV (Promega, Madison, WI). Validated qPCR primers (BLAST, National Center for Biotechnology Information (NCBI), Bethesda, MD, USA) were used and a triplicate was performed. The mRNA level of β-actin was used as a housekeeping gene, and the relative mRNA levels were determined by using the 2-ΔΔCt method. The primer sequences are listed below: PUMA forward: 5′-GACCTCAACGCACAGTACGA; reverse: 5′-ACATGGTGCAGAGAAAGTCC; NOXA forward: 5′-GTGCCCTTGGAAACGGAAGA; reverse: 5′-CAGCCGCCCAGTCTAATCA; ACTB forward: 5′-CACACCCGCCACCAGTTCGC-3′, reverse: 5′-TTGCACATGCCGGAGCCGTT-3′.

### 2.10. Statistical Analysis

The results are presented as means ± S.D. Student’s *t*-test was applied in case of comparison of only two variables, and one-way ANOVA with post hoc Tukey for multiple comparisons. GraphPad Prism 7.05 (Windows) (Boston, MA, USA) software was used. Differences were considered to be significant at *p* < 0.05.

## 3. Results

### 3.1. Treatment with T. borchii Induced a Proliferation Arrest of HCT 116 Cells

**Figure 1:**
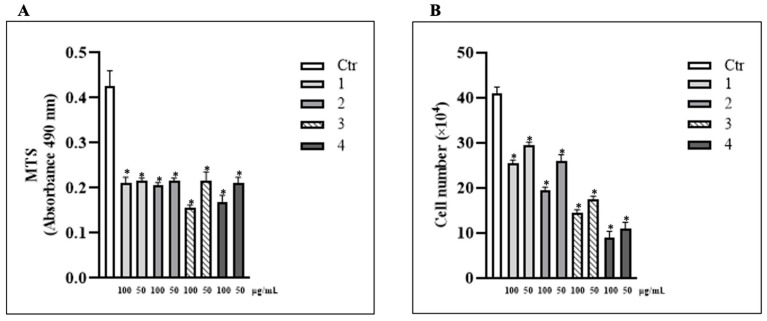
***T. borchii* extracts induce HCT 116 cells’ proliferation arrest.** (**A**) Cell proliferation was evaluated by MTS, 24 h after treatment with the four extracts of *T. borchii* (1, 2, 3 and 4) at 100 and 50 µg/mL. The data are expressed as means ± S.D. (*n* = 10; * *p* < 0.05). (**B**) Cell number was determined by Trypan Blue exclusion assay 24 h after treatment with *T. borchii* extracts (1, 2, 3 and 4) at 100 and 50 µg/mL. The data are expressed as means ± S.D. (*n* = 4; * *p* < 0.05).

In recent years, several studies have reported that truffles can synthesize numerous secondary metabolites (phenols, fatty acids, sterols, polyphenols, and antioxidants) responsible for multiple beneficial effects, including anti-inflammatory, antioxidant, and antitumor activities. These metabolites, present in various truffle species, can neutralize free radicals and protect normal cells from oxidative stress. In contrast, they can induce apoptosis in cancer cells, highlighting their potential therapeutic applications, including in chemotherapy [19]. In this study, we evaluated the effects of four extracts from different *T. borchii* truffles on the proliferation rate and viability of human HCT 116 colorectal cancer cells. Cells were treated with four extracts derived from different truffles (referred as extracts 1, 2, 3, and 4), at concentrations of 100 and 50 μg/mL. Cell proliferation was assessed using the MTS assay after 24 h. As shown in Figure 1A, all extracts significantly reduced cell proliferation at both concentrations, with extracts 3 and 4 showing the strongest inhibitory effect at 100 μg/mL.

The effects of the four extracts on HCT 116 cells proliferation were further confirmed by direct cell counts following Trypan Blue exclusion. Cell number decreased after treatment with all extracts at both concentrations, particularly for extracts 3 and 4 at 100 μg/mL after 24 h (Figure 1B). Based on these preliminary findings, we selected a concentration of 100 μg/mL for all subsequent experiments.

### 3.2. T. borchii Extracts Induce ERK1/2 Phosphorylation in HCT 116 Cells

**Figure 2:**
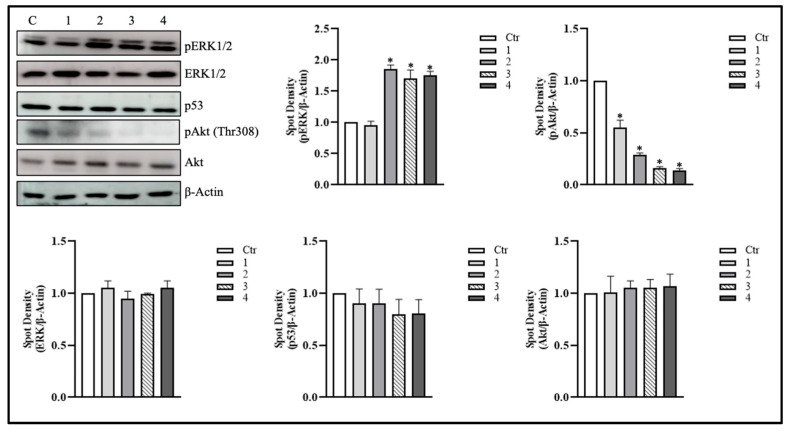
***T. borchii* evoked cell proliferation arrest was associated with activation of pERK1/2**. HCT 116 cells were treated with 100 µg/mL of extracts of *T. borchii* (1, 2, 3 and 4) for 6 h. pERK1/2, pAkt (Thr308), p53, ERK1/2 and Akt were detected by Western blotting on total protein extracts. The immunoblots reported are representative of four experiments that gave similar results. β-Actin was used as a loading control. The software Quantity One (Bio-Rad) was used to calculate density of immunoreactive bands. The data are shown as a ratio of protein/β-Actin. The data are expressed as means ± S.D. (*n* = 3; * *p* < 0.05).

To investigate the signaling pathways through which *T. borchii* extracts induce proliferation arrest in HCT 116 cells, we examined their ability to modulate key regulators of cell growth and apoptosis. Cells were treated with truffle extracts for 6 h, a time window selected to capture early signaling events underlying the observed reduction in cell proliferation. Given the established role of ERK1/2, a MAPK whose sustained activation can promote antiproliferative and pro-apoptotic outcomes [20,21], we assessed whether the extracts influenced its phosphorylation status. As shown in Figure 2, extracts 2, 3, and 4 induced a marked increase in ERK1/2 phosphorylation 6 h after treatment. In contrast, the levels of total ERK1/2 remained constant at all time points examined, indicating that the extracts specifically enhanced ERK1/2 activation rather than altering its overall expression (Figure 2).

We next analyzed Akt/PKB levels, that is another kinase implicated in the regulation of cell proliferation and apoptosis [22]. Phosphorylation of Akt (pAkt) typically inhibits apoptosis and promotes cell survival. In certain contexts, ERK activation can negatively regulate Akt activation, leading to cell death; this often occurs when ERK is overactivated in response to DNA damage or other cellular stressors [23,24]. To explore whether Akt/PKB participates in this process, we monitored its phosphorylation at key residues, which serve as markers of its activation [25]. Western blot analysis revealed that pAkt was not activated following extract treatment, as phosphorylation at Thr308 was absent, particularly in extracts 2, 3, and 4 at 6 h, suggesting that pERK1/2 activation can determine its shutdown (Figure 2). Subsequently, we analyzed the levels of p53 protein, whose activation and nuclear translocation appear to be linked to the activation of pERK1/2 in response to an apoptotic stimulus [26,27]. As shown in Figure 2, total p53 levels were not modulated by any *T. borchii* extracts after 6 h.

### 3.3. T. borchii Induces Apoptotic Cell Death in HCT 116 Cells

**Figure 3:**
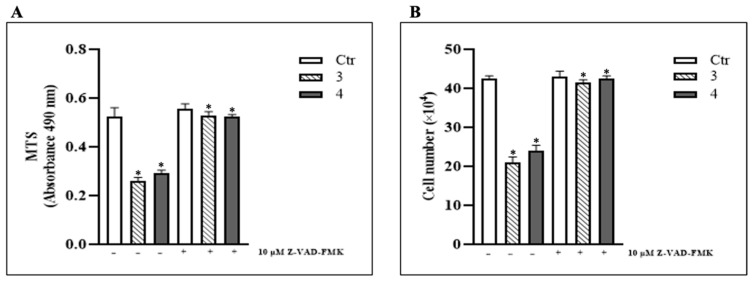
***T. borchii* extracts induce apoptosis in HCT 116 cells.** 10 µM Z-VAD-FMK were added to the culture medium 1 h prior to the addiction of *T. borchii* extracts (3 and 4) and maintained throughout the experiment. (**A**) Cell proliferation was assayed by MTS, 24 h after treatment with *T. borchii* extracts (3 and 4) at 100 µg/mL. The data are expressed as means ± S.D. (*n* = 8; * *p* < 0.05 with respect to cells treated with *T. borchii* extracts 3 and 4 alone). (**B**) Cell number was determined by Trypan Blue exclusion test 24 h after treatment with the four *T. borchii* extracts (3 and 4) at 100 µg/mL. The data are expressed as means ± S.D. (*n* = 3; * *p* < 0.05 with respect to cells treated with *T. borchii* extracts 3 and 4 alone).

To further investigate the signaling pathway responsible for cell proliferation arrest and potential apoptotic death following *T. borchii* treatment in HCT 116 cells, we evaluated the effect of Z-VAD-FMK. This caspase inhibitor irreversibly binds to the active site of caspases, blocking their enzymatic activity and thereby preventing activation of the classical apoptotic cascade. For the subsequent experiments, we selected *T. borchii* extracts 3 and 4, both used at a concentration of 100 μg/mL, since these extracts exhibited the most pronounced effects on proliferation arrest and pERK1/2 activation. Cell quantification performed using the Trypan Blue exclusion assay, together with MTS viability analysis (Figure 3A,B), showed that treatment with 10 μM Z-VAD-FMK completely abolished the antiproliferative activity of extracts 3 and 4.

These findings indicate that *T. borchii* extracts trigger apoptosis in HCT 116 colorectal cancer cells following 24 h of treatment.

**Figure 4:**
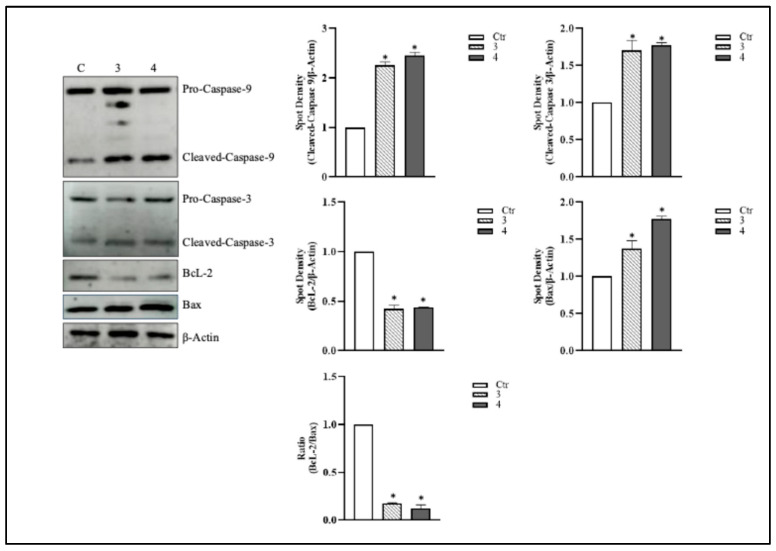
***T. borchii* induces apoptosis through activation of the mitochondrial pathway.** HCT 116 cells were treated with 100 µg/mL of *T. borchii* extracts (3 and 4) for 24 h. Caspase-9, caspase-3, BcL-2 and Bax levels were detected by Western blotting on total protein extracts. The immunoblots reported are representative of four experiments that gave similar results. β-Actin was used as a loading control. The software Quantity One (Bio-Rad) was used to calculate density of immunoreactive bands. The data are shown as a ratio of protein/β-Actin or a ratio of BcL-2/Bax. The data are expressed as means ± S.D. (*n* = 4; * *p* < 0.05).

We next examined the activation status of caspase-3 and caspase-9, assessing both their procaspase (inactive) and cleaved (active) forms, a widely accepted method for confirming apoptosis induction [28,29]. Following treatment of HCT 116 cells with *T. borchii* extracts 3 and 4 (100 µg/mL for 24 h), we observed a marked increase in the cleaved forms of caspase-9 and caspase-3, indicating robust activation of the intrinsic apoptotic pathway (Figure 4).

**Figure 5:**
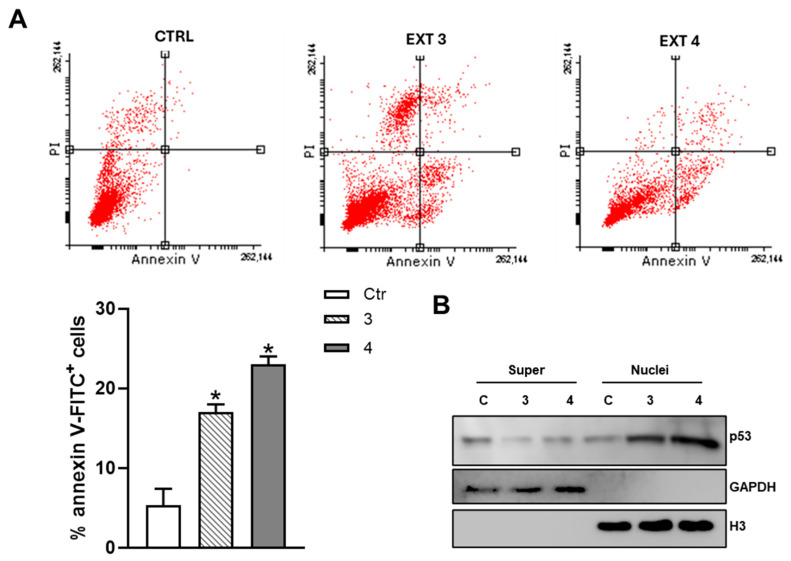
***T. borchii* treatment promotes cell death through the translocation of p53 into the nuclei of HCT 116 cells.** (**A**) HCT 116 cells were treated with the extracts of *T. borchii* (3 and 4) at 100 µg/mL for 24 h and stained with annexin V-FITC and propidium iodide (flow cytometry dot plots). Fluorescence was analyzed by a FACScalibur instrument. The percentages of staining-positive cells were evaluated using WinMDI version 2.8 software. The data show the percentage of total annexin V-FITC-positive cells and expressed as means ± S.D. (*n* = 3; * *p* < 0.05). (**B**) HCT 166 cells were treated with 100 µg/mL extracts 3 and 4 of *T. borchii* for 6 h. p53 protein content was detected by Western blot on supernatant and nuclear protein extracts. The immunoblots reported are representative of four experiments that gave similar results. GAPDH and H3 were used as purity controls.

This increase confirms that *T. borchii* treatment activates the apoptotic pathway: caspase-9 is likely cleaved downstream of mitochondrial dysfunction and cytochrome *c* release, subsequently activating caspase-3, the final executor of apoptosis. The detection of cleaved caspase-3 and caspase-9 in our experimental conditions provides strong evidence that *T. borchii* extracts not only enhance caspase expression but also promote their proteolytic activation, a critical step in programmed cell death. Furthermore, we analyzed levels of the anti-apoptotic protein BcL-2, a mitochondrial factor associated with apoptosis [30,31]. Figure 4 shows that treatment with 100 μg/mL of *T. borchii* extracts 3 and 4 for 24 h resulted in a reduction in BcL-2 expression and a concomitant increase in Bax in HCT 116 cells. This modulation of Bcl-2 family proteins indicates activation of the intrinsic apoptotic pathway, most likely driven by an increased Bax/Bcl-2 ratio—a well-recognized marker of cellular susceptibility to apoptosis. These findings support the notion that the bioactive compounds contained in truffle extracts may exert antitumor properties by enhancing mitochondrial-mediated programmed cell death. These findings were further supported by FACS analysis. HCT 116 cells were treated with 100 μg/mL of extracts 3 and 4 for 24 h and then analyzed by flow cytometry after staining with annexin V-FITC/propidium iodide. As shown in Figure 5, the percentages of annexin V-FITC/propidium iodide-positive cells were consistent with the Western blot, MTS analysis and Trypan blue exclusion assay data (see Figure 3 and Figure 4).

### 3.4. T. borchii Extracts Induce Nuclear Translocation of p53 and Up-Regulation of Its Pro-Apoptotic Targets PUMA and NOXA in HCT 116 Cells

p53 is one of the key transcription factors downstream of ERK1/2 involved in regulating both cell-cycle progression and apoptotic cell death [26,32,33]. The involvement of p53 was investigated by monitoring its nuclear translocation, as no significant changes were observed in its total protein levels (see Figure 2). We then isolated cell nuclei and assessed p53 content by Western blot analysis. As shown in Figure 5, treatment of HCT 116 cells with *T. borchii* extracts 3 and 4 induced a clear nuclear translocation of p53 after 6 h, indicating that apoptosis in these cells is likely mediated through the pERK1/2–p53 signaling axis.

**Figure 6:**
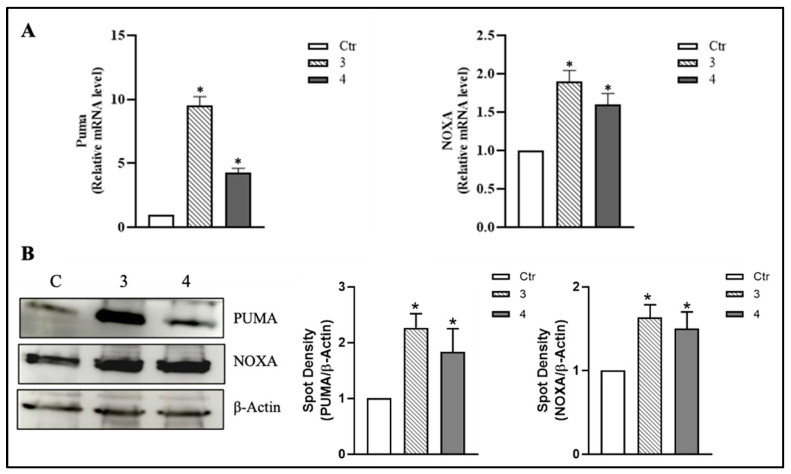
**Treatment with *T. borchii* extracts leads to the upregulation of the pro-apoptotic proteins PUMA and NOXA in HCT 116 cells.** (**A**) Relative mRNA levels of PUMA and NOXA were analyzed by RT-qPCR. The data are expressed as means ± S.D. (*n* = 4; * *p* < 0.05 vs. control). (**B**) HCT 116 cells were treated with 100 µg/mL of *T. borchii* extracts (3 and 4) for 24 h. PUMA and NOXA levels were detected by Western blotting on total protein extracts. The immunoblots reported are representative of four experiments that gave similar results. β-Actin was used as a loading control. The software Quantity One (Bio-Rad) was used to calculate density of immunoreactive bands. The data are shown as a ratio of protein/β-Actin. The data are expressed as means ± S.D. (*n* = 3; * *p* < 0.05).

To further establish that p53 nuclear translocation triggered by *T. borchii* extracts results in the activation of the apoptotic program, we examined the expression of two well-characterized p53-dependent pro-apoptotic factors, PUMA and NOXA [34]. PUMA and NOXA mRNA levels were analyzed by RT-qPCR, and, as shown in Figure 6A, treatment with *T. borchii* extracts 3 and 4 resulted in significant induction of their mRNA expression at 12 h. Next, we analyzed whether the protein content of PUMA and NOXA could be modulated after treatment with *T. borchii* extracts 3 and 4. Western blot analysis showed an increase in the protein content of PUMA and NOXA in the total extracts of HCT 116 cells after 24 h of treatment (Figure 6B).

These findings demonstrate that the p53 nuclear translocation triggered by the extracts is functionally active, leading to the transcriptional upregulation of its pro-apoptotic targets, PUMA and NOXA. Collectively, these data confirm that apoptosis induction in HCT 116 cells occurs through the pERK1/2–p53 signaling axis.

## 4. Discussion

CRC is one of the most prevalent malignancies globally and remains a leading cause of cancer-related mortality [1]. A complex interplay of genetic factors and modifiable lifestyle components drives its onset and progression. Among these, diet has emerged as a critical determinant, influencing both the risk of tumor development and the clinical trajectory of the disease [35].

The colorectal epithelium is in constant contact with dietary components, including not only nutrients but also potentially bioactive compounds. This direct exposure renders CRC particularly sensitive to dietary influences. On the one hand, an unbalanced diet—especially one high in saturated fats—has been strongly linked to an increased risk of CRC. On the other hand, this same interface offers a strategic advantage: it enables the exploration and use of orally administered bioactive molecules capable of exerting preventive or therapeutic effects on colorectal tumorigenesis.

As such, CRC represents an ideal model for investigating the impact of diet-derived compounds and nutraceuticals on cancer biology, offering valuable insights into the development of novel, non-invasive interventions that modulate disease outcomes through dietary modulation.

More recently, natural compounds have gained increasing attention as adjuvants to conventional cancer therapies due to their multiple advantages, foremost among them their low or negligible toxicity. Several of these molecules, when tested on CRC models, have demonstrated the ability to inhibit cell proliferation by modulating the cell cycle and promoting apoptosis [36].

Based on this rationale, the present study investigated in vitro the effects of truffle (*Tuber borchii*) extracts on colorectal cancer cells. Previous studies have reported that truffle extracts possess a wide range of biological activities, notably antioxidant and anti-inflammatory effects [37,38,39]. Indeed, truffles are rich in various antioxidant compounds, including ascorbic acid, ergosterol, phenolics, flavonoids, terpenoids, phytosterols, and polysaccharides, all of which have been extensively studied for their antioxidant capacity. These bioactive molecules have shown promise as potential adjuvants in cancer therapy, due to their ability to modulate oxidative stress and inflammatory responses, both critical components in cancer development and progression [7,40]. Moreover, these properties led us to hypothesize that truffle extracts may exert not only direct anticancer effects on tumor cells, but also modulatory effects on the tumor microenvironment. In particular, many nutraceuticals that have shown beneficial effects in CRC also exhibit antioxidant properties, which appear to be a crucial factor in this context. Oxidative stress has been identified as a key driver in colorectal tumorigenesis, not only initiating malignant transformation but also sustaining chronic inflammation, which further promotes tumor progression [41].

Given that the ability of truffle extracts to inhibit tumor cell proliferation is well documented in the literature [42,43], in the present study we tested, for the first time, various *T. borchii* extracts in vitro on HCT 116 colorectal cancer cells. *T. borchii* is an edible ectomycorrhizal mushroom of considerable economic and biological relevance [44]. It naturally grows throughout Europe and can thrive under suboptimal environmental conditions, making it a desiderable species for biotechnological and therapeutic applications. The identification of nutraceutical and antitumor properties in *T. borchii* further enhances its significance, expanding its value beyond gastronomy toward potential biomedical exploitation.

Our findings demonstrate that these extracts can reduce cell proliferation and induce cytotoxicity in CRC cells. Moreover, we demonstrated that the cytotoxic effect of *T. borchii* extracts was mediated by activation of pro-apoptotic pathways, as evidenced by increased cleavage of caspase-3 and caspase-9, along with a decreased Bcl-2/BAX ratio, indicating enhanced susceptibility to apoptosis in treated cells. In CRC, the Bcl-2/BAX ratio has been identified as a crucial prognostic marker, correlating with tumor grade, stage, and size in patients. Consequently, this ratio has significant prognostic value, as it predicts of patient survival, tumor relapse, and response to chemotherapeutic agents [45].

The complete abrogation of the cytotoxic effect upon co-treatment with the pan-caspase inhibitor Z-VAD-FMK confirmed the activation of apoptosis upon truffle extracts treatment.

Consistently, we observed an increased transcription of pro-apoptotic genes such as PUMA and NOXA. Although these genes are downstream targets of the transcription factor p53 [46], which typically responds to pro-apoptotic stimuli, total p53 levels remained unchanged following treatment with truffle extracts. For this reason, we performed a nuclear extraction and demonstrated that, although total p53 levels remained unchanged, p53 was more abundant in the nuclei of cells treated with truffle extracts compared to control cells, indicating increased p53 transcriptional activity.

Therefore, to further elucidate the mechanism underlying apoptosis induction mediated by the extracts, we investigated the activation of the ERK and AKT signaling pathways. Indeed, the literature demonstrates that many natural compounds modulate cancer cell proliferation by regulating ERK signaling pathway [47].

The effects of ERK activation vary depending on the cell type and external stimuli, leading to different antiproliferative outcomes such as apoptosis, autophagy, or senescence in both in vitro and in vivo settings. ERK signaling can trigger apoptotic pathways by promoting mitochondrial cytochrome *c* release or activating caspase-8 [48].

Specifically, a recent study has identified an antiproliferative role of ERK in the intestinal epithelium [49]. Moreover, the reduction in AKT activation aligns well with the increased expression of BAX and decreased expression of BcL-2, collectively directing the cell toward apoptosis [50]. Indeed, the reduction in AKT activation, demonstrated here as decreased phosphorylation at Thr308, represents a key target in the development of new anticancer drugs aimed at overcoming therapeutic resistance [51].

## 5. Conclusions

In conclusion, these data demonstrate that extracts derived from *T. borchii* exert cytotoxic effects on colorectal cancer cells by inducing apoptosis, even basally in the absence of additional chemotherapeutic agents. Overall, this study lays the groundwork for further exploration of *T. borchii* extracts as a potential nutraceutical, adjuvant to conventional cancer therapy.

Given the high prevalence of CRC and the substantial likelihood that resistance to conventional therapies may develop, the identification of natural extracts with pro-apoptotic activity is of particular interest. Indeed, the aim is not only to identify potential adjuvants to CRC chemotherapy, but also to reduce the required doses of chemotherapeutic agents, thereby limiting the emergence of resistance.

## Figures and Tables

**Table 1:** Summary table of the antibodies used, with code and brand.

Antibody	Code	Brand
Akt	9272	Cell Signaling
Bax	2772	Cell Signaling
BcL-2	B3170	Sigma-Aldrich
β-actin	4970S	Cell Signaling
Caspase-3	C8487	Sigma-Aldrich
Caspase-9	05-572	Upstate
ERK 1/2	9102	Cell Signaling
GAPDH	2118	Cell Signaling
Histone H3	9715	Cell Signaling
p53	P5813	Sigma-Aldrich
p-Akt ^1^/_2_ (Thr 308)	9275	Cell Signaling
p-ERK 1/2	9101	Cell Signaling

## Data Availability

The datasets used and analyzed during the current study are available upon request from the corresponding author.

## Supplementary Materials

The supporting information can be downloaded at https://www.mdpi.com/article/10.3390/metabo15120796/s1. Supplementary Figure S1. Microscopic observation of the gleba of Tuber borchii fruiting bodies selected for the analyses (a: extracts 1, b: extracts 2, c: extracts 3, and d: extracts 4), all showing a maturation degree of 1. Materials and Methods. The maturation stage of the fruiting bodies was evaluated following the method described by Zeppa et al. (2004) [17]. Only ascomata exhibiting a maturation degree of 1, corresponding to 6–30% of mature spores, were selected for analysis (Supplementary Figure S1).

## Abbreviations

The following abbreviations are used in this manuscript:

Akt	Protein kinase B
BAX	Bcl-2 Associated X-protein
Bcl-2	B-cell lymphoma 2
CRC	Colorectal cancer
ERK1/2	Extracellular signal-Regulated Kinases1/2
GAPDH	Glyceraldehyde 3-phosphate dehydrogenase
NOXA	Phorbol-12-myristate-13-acetate-induced protein 1
PUMA	p53 upregulated modulator of apoptosis
Z-VAD-FMK	Carbobenzoxy-valyl-alanes-aspartyl-(O-methyl)-fluoromethylketone
