# Supplementary material for: Antiproliferative and Pro-Apoptotic Effects of Tuber borchii Extracts on Human Colorectal Cancer Cells via p53-Dependent Pathway Activation
Source: Metabolites. 2025 Dec 15;15(12):796. doi: 10.3390/metabo15120796 (PMC12735153; doi:10.3390/metabo15120796)
Supplement: Supplementary file 1 [file metabolites-15-00796-s001.zip › metabolites-4014241-supplementary.pdf]

Supplementary Figure S1

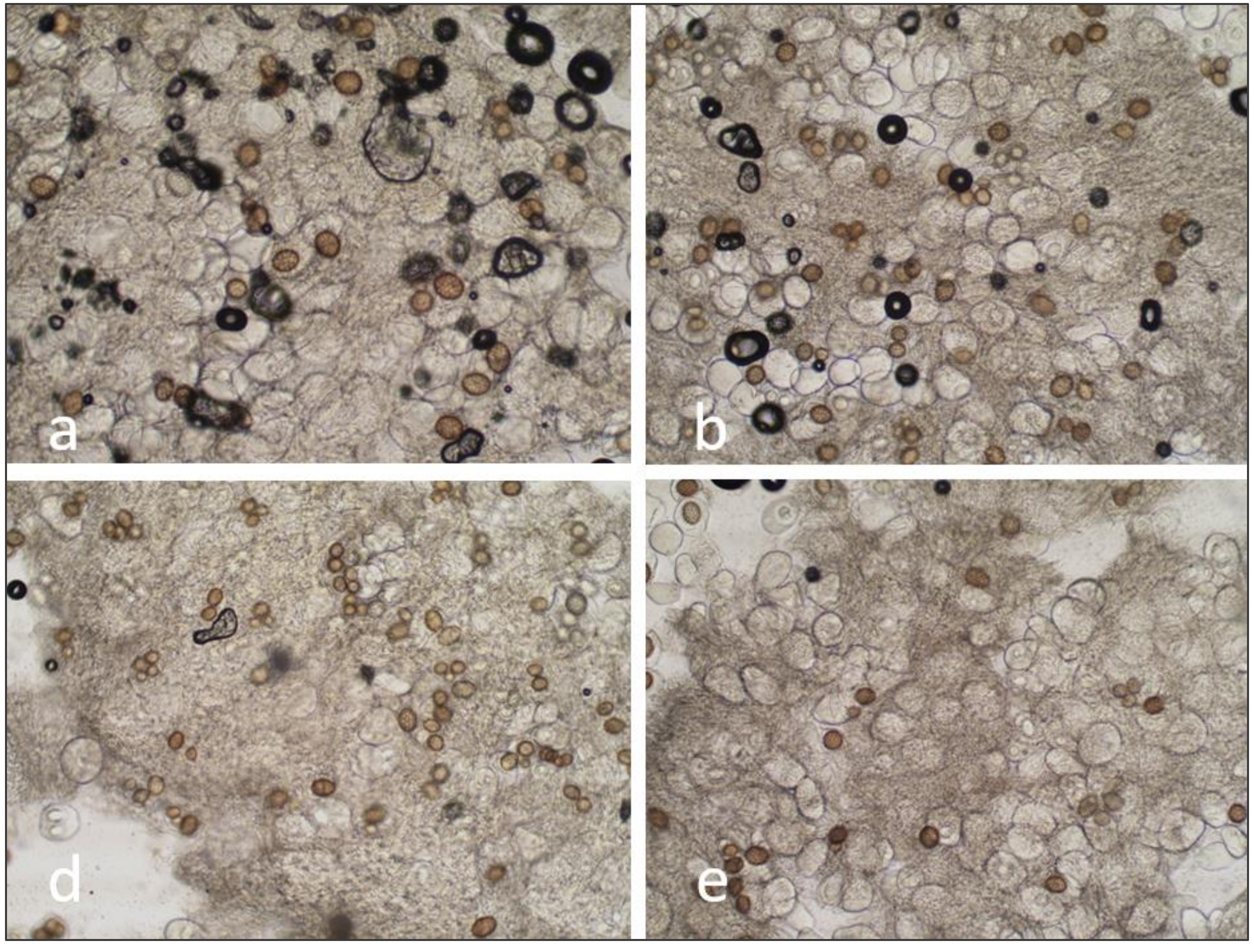

Supplementary Figure S1. Microscopic observation of the gleba of *Tuber borchii* fruiting bodies selected for the analyses (a: extracts 1, b: extracts 2, d: extracts 3 and e: extracts 4), all showing a maturation degree of 1.
